# Inferior Hip Dislocation in a 60-Year-Old Man; a Case Report

**DOI:** 10.22037/aaem.v10i1.1498

**Published:** 2022-02-27

**Authors:** Ali Yeganeh, Nader Tavakoli, Mohammad Soleimani, Seyed Nima Taheri, Sahand Cheraghiloohesara

**Affiliations:** 1 Trauma and Injury Research Center, School of Medicine, Iran University of Medical Sciences, Tehran, Iran.; 2 Department of Epidemiology, School of Public Health, Iran University of Medical Sciences, Tehran, Iran.

**Keywords:** Hip dislocation, joint dislocations, case reports, Wounds and Injuries

## Abstract

Inferior hip dislocation or luxatio erecta femoris is among the rarest hip dislocations, which has been described in limited studies. The patients usually present with their hip in flexion, abduction, and external rotation. Hip dislocation is an orthopedic emergency, and a reduction needs to be performed promptly to avoid devastating complications such as avascular necrosis. Here, we present a rare case of inferior hip dislocation in a 60-year-old man following a car-motorcycle collision. The patient presented to the emergency department with left hip flexion, abduction, external rotation, and inability to move his leg due to pain. Closed reduction under procedural sedation was attempted in the emergency department once, which was unsuccessful. The patient was then taken to the operating room for another attempt of closed reduction under general anesthesia. The patient was discharged after two days with pin traction and double crutches. After two weeks, the pin was removed, and full weight-bearing was permitted. After 12 weeks, the patient had mild pain with unusual activity and slight limping; however, imaging revealed no signs of any complications.

## 1. Introduction

As the most stable joint, the hip requires much energy to be dislocated; so, hip dislocations comprise 5% of all joint dislocations (1). Not surprisingly, most hip dislocations are due to high-energy trauma. Hip dislocations are categorized into three main groups: posterior, central, and anterior (2). The most frequently observed subtype of hip dislocations is posterior dislocation, whereas anterior dislocation is the rarest, comprising less than 10% of all hip dislocations (1). Anterior dislocations are further categorized into superior and inferior dislocation or luxatio erecta femoris (3, 4). Inferior hip dislocation comprises 5% of all traumatic hip dislocations. (1, 5, 6).

Here, we present a rare case of inferior hip dislocation with no concomitant injury or fracture, managed through closed reduction.

## 2. Case Presentation

A 60-year-old man presented to our emergency department following a car vs. motorcycle collision, in which the patient was the biker. He was brought to our hospital by the emergency medical service. Upon arrival, the patient was complaining of pain, his left hip was in flexion, abduction, and external rotation, and he was unable to move the leg.

Apart from skin abrasions and deformity in the left hip, the primary survey was unremarkable. In the secondary survey, there was tenderness in the left hip, limited range of motion, no neurologic deficits, and normal distal pulses of the limb. The patient recalled the incident and complained of constant severe pain in the left groin after the incident, which worsened by limb movement.

**Figure 1:**
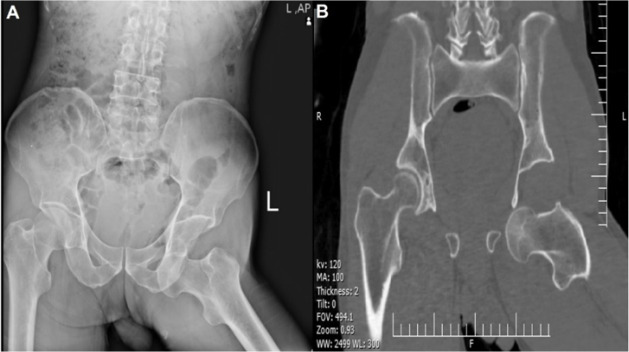
(A) Primary pelvic x-ray radiograph, (B) Pre-reduction computed tomography (CT) scan

A radiograph showed inferior hip dislocation (Figure 1A). A computed tomography (CT) scan confirmed the diagnosis and showed no sign of fracture (Figure 1B). Physical examination and imaging revealed no other injuries than hip dislocation. 

Closed reduction under sedation with propofol and midazolam was tried in the emergency department, which was unsuccessful.

**Figure 2:**
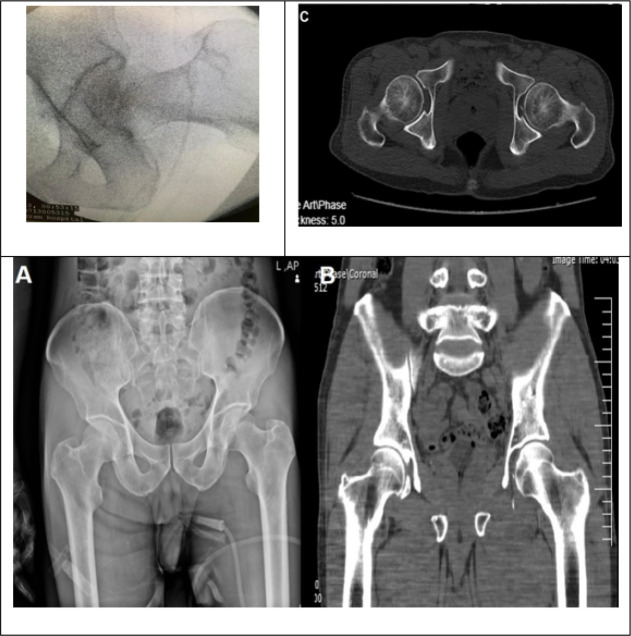
Reduction confirmed by fluoroscopy in the operation room, pelvic X-ray radiograph (A), and coronal (B) and axial (C) views of pelvic computed tomography scan

Next, the patient was taken to the operating room for another attempt at closed reduction under general anesthesia. The patient lied in the supine position on the operating table, and the hip was reduced in the first attempt using the Allis maneuver. Then, the range of motion was evaluated, which was normal, and the hip was stable. The concentric reduction was confirmed via fluoroscopy in the operating room, and post-operative pelvic radiograph and computed tomography (CT) scan (Figure 2).

After the reduction, pin traction and a knee immobilizer were used for the patient. Then he was admitted to the orthopedic surgery ward and was on complete bed rest for the next two days until discharge. Upon discharge, he was instructed on partial weight-bearing on the injured limb and using double crutches for the next two weeks.

**Figure 3:**
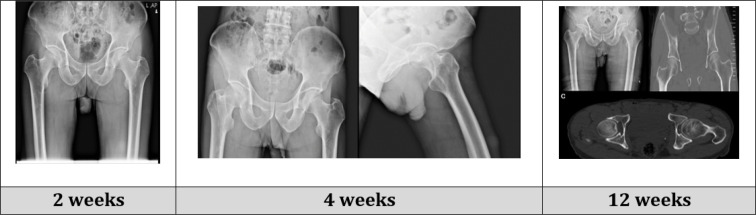
Follow-up imaging of patient at various times after reduction

Figure 3 shows the results of the patient’s pelvic imaging during the 12-week follow-up. Two weeks later, the patient presented to the hip clinic for his first follow-up. He had no complaint of pain. In physical examination, the range of motion was normal, and the hip was stable. Also, there was no tenderness in the left hip. The pin traction was removed, and full-weight bearing was permitted. 

For his subsequent follow-up, the patient presented to the clinic after another two weeks and reported mild pain and returning to normal daily activity without any difficulty. In the physical examination, he had slight limping in gait, a normal range of motion of the hip, and no tenderness. Also, the imaging showed no sign of any complications.

Two months later, 12 weeks after the reduction, the patient returned for his next appointment. He reported mild pain with unusual activities, and the physical examination indicated slight limping in the gait, while palpitation, range of motion, and stability of the joint were normal. A Harris Hip Score of 73 was calculated for the patient. Pelvic radiograph and CT scan were obtained, which showed no signs of any complications.

## 3. Discussion

Bigelow first described traumatic hip dislocation in 1869, since then, hip dislocation’s main injury pattern has changed from horseback riding to motor vehicle accidents (7). These injuries mostly affect young men and result from a motor vehicle accident in up to 93% of cases. Other causes include pedestrian accidents, falls, and sports (1, 3, 7).

Since the hip is a very stable joint due to the surrounding muscle and ligament structures that provide protection, hip dislocations mainly occur following high-energy trauma (1, 7, 8). However, in children, these injuries generally occur after low-energy trauma due to the acetabulum structure and laxity of joints (9).

Hip dislocation comprises 5% of all joint dislocations and is categorized into the anterior, central, and posterior main groups (2). Posterior dislocation is the most common subtype, and anterior dislocation is the rarest, and the ratio of posterior to anterior dislocations is reported to be between 10:1 to 19:1 (7). Anterior dislocation is itself divided into superior and inferior dislocations (2-4, 10). Inferior hip dislocations are responsible for almost 5% of all traumatic hip dislocations (1, 5, 6).

Ocurrence of a superior or inferior dislocation depends on the position of the hip at the time of trauma. Trauma to a hip in extension, abduction, and external rotation results in superior dislocation, while trauma to a flexed, abducted, and externally rotated hip results in inferior hip dislocation (7).

Hip dislocation is an orthopedic emergency, and the cornerstone of treatment is timely diagnosis and reduction (7). Also, traumatic hip dislocation is usually associated with several other traumas, including head, neck, chest, and abdomen, and femoral head or neck fractures (2, 9). Thus, obtaining pelvic radiographs is required to prevent the overlooking of possible hip dislocations (7).

Several studies indicate that the treatment of choice is closed reduction if there is no sign of fracture or loose fragments (5, 9-12). However, several attempts on the closed reduction may itself lead to complications and is contraindicated, as the success rates of open and closed reduction are reported to be the same (1, 7).

There is no consensus over the post-reduction management (7). Older studies prefer prolonged immobilization, prevention of weight-bearing, and traction. However, no studies support these methods, and a shorter period of immobilization and non-weight-bearing may result in the same outcomes (3, 7).

Hip dislocation is an orthopedic emergency and can lead to several complications (5). A terrifying complication of hip dislocation is avascular necrosis (AVN) of the femoral head (5, 13). Jacob et al. indicate a significant reduction in AVN with prompt diagnosis and management, i.e., within the first six hours; thus, promptly reducing the dislocated hip is crucial (7, 10, 14).

Other significant complications of hip dislocation include arthritis, nerve injury, and myositis ossificans, and the patient should be followed to promptly diagnose and manage these complications (11).

## 4. Conclusion:

Hip dislocation is rare and usually occurs following high-energy traumas, and inferior dislocation is among the rarest. These injuries may result in AVN and arthritis, and it is crucial to promptly diagnose and manage them within the first six hours to avoid complications.

## 5. Declarations:

### 5.1. Authors’ contributions

NT met the patient in the emergency department. AY, NT, and SC reduced the hip in the operating room. NT and AY suggested the concept of work. MS, NT, and SC made data acquisition. MS wrote the first draft, which was read and revised by all others. All authors approved the final version for submission and agreed to be accountable for all aspects of the work.

### 5.2. Funding and support

None

### 5.3. Conflict of interest

None

### 5.4. Data availability

Authors guarantee that data of the study is available and will be provided to anyone needing it.

### 5.5. Ethical issues

Written informed consent was obtained from the patient, and the study was approved by the Ethics Committee of the Iran University of Medical Sciences under the code IR.IUMS.REC.1400.765.

### 5.6 Acknowledgment

We would like to thank the patient for granting us permission to use his data. Also, we would like to thank Dr. Amir Sobhani Eraghi and Dr. Masoud Aslani for their contributions to this study.
